# 6,14-Dibromo-2,11-dithia­[3.3]paracyclo­phane

**DOI:** 10.1107/S1600536810028874

**Published:** 2010-07-24

**Authors:** Xingxun Zhu, Ming Hu

**Affiliations:** a Key Laboratory of Pesticides and Chemical Biology of the Ministry of Education, College of Chemistry, Central China Normal University, Wuhan 430079, People’s Republic of China

## Abstract

In the title compound, C_16_H_14_Br_2_S_2_ [systematic name: 1^2^,5^2^-dibromo-2,7-dithia-1,5(1,4)-dibenzenaocta­phane], the cen­troids of the two benzene rings are separated by 3.313 (5) Å. The crystal packing exhibits weak inter­molecular S⋯S contacts of 3.538 (2) Å.

## Related literature

For the preparation of the title compound, see: Wang *et al.* (2003 ▶, 2006 ▶). For a related structure, see: Huang *et al.* (2010 ▶).
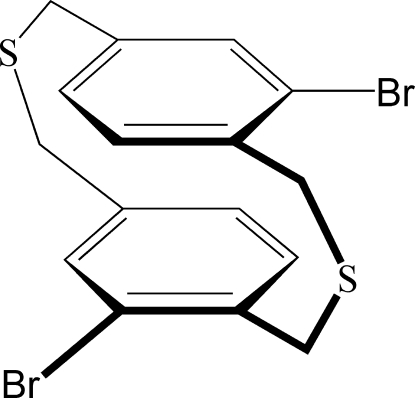


## Experimental

### 

#### Crystal data

C_16_H_14_Br_2_S_2_
*M* *_r_* = 430.21
Orthorhombic, 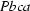

*a* = 9.0563 (11) Å
*b* = 13.8931 (17) Å
*c* = 24.641 (3) Å
*V* = 3100.4 (7) Å^3^
*Z* = 8
Mo *K*α radiation
μ = 5.49 mm^−1^
*T* = 298 K
0.26 × 0.20 × 0.10 mm

#### Data collection

Bruker SMART APEX diffractometer
Absorption correction: multi-scan (*SADABS*; Sheldrick, 1996 ▶) *T*_min_ = 0.330, *T*_max_ = 0.610
21950 measured reflections
3372 independent reflections
2150 reflections with *I* > 2σ(*I*)
*R*_int_ = 0.12

#### Refinement

*R*[*F* ^2^ > 2σ(*F* ^2^)] = 0.041
*wR*(*F* ^2^) = 0.115
*S* = 0.99
3372 reflections
181 parameters
H-atom parameters constrained
Δρ_max_ = 0.93 e Å^−3^
Δρ_min_ = −0.38 e Å^−3^

### 

Data collection: *SMART* (Bruker, 2007 ▶); cell refinement: *SAINT* (Bruker, 2007 ▶); data reduction: *SAINT*; program(s) used to solve structure: *SHELXS97* (Sheldrick, 2008 ▶); program(s) used to refine structure: *SHELXL97* (Sheldrick, 2008 ▶); molecular graphics: *SHELXTL* (Sheldrick, 2008 ▶); software used to prepare material for publication: *SHELXTL*.

## Supplementary Material

Crystal structure: contains datablocks I, global. DOI: 10.1107/S1600536810028874/cv2739sup1.cif

Structure factors: contains datablocks I. DOI: 10.1107/S1600536810028874/cv2739Isup2.hkl

Additional supplementary materials:  crystallographic information; 3D view; checkCIF report

## Figures and Tables

**Fig. 1.**
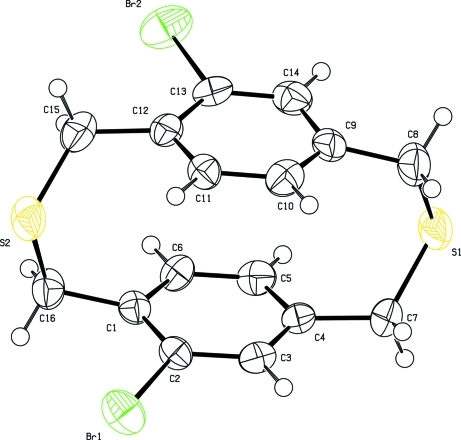
A view of (I), showing the atom-labelling scheme, with displacement ellipsoids drawn at the 50% probability level.

## supplementary crystallographic information

### Comment

As a contribution to a structural study of dithia[3.3]paracyclophane derivatives with the bromine sustituents (Huang *et al.*, 2010), herewith we present the crystal structure of the title compound (Fig. 1).

The short distance of 3.313 (5) Å between the centroids of two benzene rings  is less than the normal packing distance (3.4 Å) between the aromatic rings in organic  compounds, thus supporting potential transannular π-π interaction between the rings in the cyclophane unit.

### Experimental

The dithiaparacyclophanes were prepared by coupling the corresponding pair of dithiol and dibromide under high dilution conditions (Wang *et al.*, 2003, 2006). A solution with equimolar amounts of the dithiol and the dibromide in degassed THF (500 ml) was added dropwise under N_2_ over 12 h to a refluxing solution of K_2_CO_3_ (5 equiv) in EtOH (1.2 *L*). After an additional 2 h at the reflux temperature, the mixture was cooled and the solvent were removed. The resulting residue was treated with CH_2_Cl_2_(300 ml) and water (300 ml).The organic phase was separated, the aqueous extracted with CH_2_Cl_2_ three times. The combined organic layers were dried over Na_2_SO_4_, then solvent was removed, and the resulting solid was chromatographed on silica gel using CH_2_Cl_2_ petroleum ether (1:1, *v*/*v*) as eluent.

### Refinement

All H atoms were initially located in a difference map, but were constrained to an idealized geometry. Constrained bond lengths and isotropic displacement parameters: (C—H =0.93 Å) and *U*_iso_(H) =1.2*U*_eq_(C) for aromatic H atoms, and (C—H =0.97 Å) and *U*_iso_(H) =1.2*U*_eq_(C) for methylene, and (C—H =0.96 Å) and *U*_iso_(H)=1.5*U*_eq_(C) for methyl.

### Crystal data

**Table:**

C_16_H_14_Br_2_S_2_	*F*(000) = 1696
*M**_r_* = 430.21	*D*_x_ = 1.839 Mg m^−^^3^
Orthorhombic, *P**b**c**a*	Mo *K*α radiation, λ = 0.71073 Å
Hall symbol: -P 2ac 2ab	Cell parameters from 5026 reflections
*a* = 9.0563 (11) Å	θ = 2.8–25.5°
*b* = 13.8931 (17) Å	µ = 5.49 mm^−^^1^
*c* = 24.641 (3) Å	*T* = 298 K
*V* = 3100.4 (7) Å^3^	Block, colourless
*Z* = 8	0.26 × 0.20 × 0.10 mm

### Data collection

**Table:**

Bruker SMART APEX diffractometer	3372 independent reflections
Radiation source: fine-focus sealed tube	2150 reflections with *I* > 2σ(*I*)
graphite	*R*_int_ = 0.12
phi and ω scans	θ_max_ = 27.0°, θ_min_ = 1.7°
Absorption correction: multi-scan (*SADABS*; Sheldrick, 1996)	*h* = −10→11
*T*_min_ = 0.330, *T*_max_ = 0.610	*k* = −17→17
21950 measured reflections	*l* = −31→31

### Refinement

**Table:**

Refinement on *F*^2^	Primary atom site location: structure-invariant direct methods
Least-squares matrix: full	Secondary atom site location: difference Fourier map
*R*[*F*^2^ > 2σ(*F*^2^)] = 0.041	Hydrogen site location: inferred from neighbouring sites
*wR*(*F*^2^) = 0.115	H-atom parameters constrained
*S* = 0.99	*w* = 1/[σ^2^(*F*_o_^2^) + (0.0521*P*)^2^] where *P* = (*F*_o_^2^ + 2*F*_c_^2^)/3
3372 reflections	(Δ/σ)_max_ = 0.001
181 parameters	Δρ_max_ = 0.93 e Å^−^^3^
0 restraints	Δρ_min_ = −0.38 e Å^−^^3^

### Special details

**Table:**

Geometry. All e.s.d.'s (except the e.s.d. in the dihedral angle between two l.s. planes) are estimated using the full covariance matrix. The cell e.s.d.'s are taken into account individually in the estimation of e.s.d.'s in distances, angles and torsion angles; correlations between e.s.d.'s in cell parameters are only used when they are defined by crystal symmetry. An approximate (isotropic) treatment of cell e.s.d.'s is used for estimating e.s.d.'s involving l.s. planes.
Refinement. Refinement of *F*^2^ against ALL reflections. The weighted *R*-factor *wR* and goodness of fit *S* are based on *F*^2^, conventional *R*-factors *R* are based on *F*, with *F* set to zero for negative *F*^2^. The threshold expression of *F*^2^ > σ(*F*^2^) is used only for calculating *R*-factors(gt) *etc*. and is not relevant to the choice of reflections for refinement. *R*-factors based on *F*^2^ are statistically about twice as large as those based on *F*, and *R*- factors based on ALL data will be even larger.

### Fractional atomic coordinates and isotropic or equivalent isotropic displacement parameters (Å^2^)

**Table:**

	*x*	*y*	*z*	*U*_iso_*/*U*_eq_	
Br1	−0.01106 (5)	0.31316 (4)	0.233829 (17)	0.05380 (18)	
Br2	−0.18330 (6)	0.72155 (4)	0.04515 (2)	0.0638 (2)	
C1	−0.0858 (4)	0.4030 (3)	0.13260 (15)	0.0325 (9)	
C2	0.0281 (4)	0.3739 (3)	0.16606 (15)	0.0324 (9)	
C3	0.1766 (4)	0.3880 (3)	0.15182 (16)	0.0364 (9)	
H3	0.2509	0.3694	0.1757	0.044*	
C4	0.2127 (4)	0.4289 (3)	0.10301 (17)	0.0364 (9)	
C5	0.0981 (4)	0.4501 (3)	0.06696 (15)	0.0397 (10)	
H5	0.1197	0.4726	0.0323	0.048*	
C6	−0.0453 (4)	0.4379 (3)	0.08239 (15)	0.0366 (9)	
H6	−0.1194	0.4540	0.0579	0.044*	
C7	0.3717 (4)	0.4516 (3)	0.0900 (2)	0.0507 (11)	
H7A	0.4112	0.3984	0.0690	0.061*	
H7B	0.4260	0.4536	0.1239	0.061*	
C8	0.3707 (5)	0.6542 (3)	0.1030 (2)	0.0527 (12)	
H8A	0.4314	0.6433	0.1348	0.063*	
H8B	0.3986	0.7160	0.0878	0.063*	
C9	0.2122 (4)	0.6589 (3)	0.12027 (16)	0.0380 (10)	
C10	0.1679 (4)	0.6251 (3)	0.16986 (17)	0.0420 (10)	
H10	0.2385	0.6104	0.1960	0.050*	
C11	0.0198 (4)	0.6126 (3)	0.18176 (16)	0.0353 (9)	
H11	−0.0071	0.5876	0.2153	0.042*	
C12	−0.0886 (4)	0.6365 (3)	0.14498 (15)	0.0342 (9)	
C13	−0.0416 (5)	0.6791 (3)	0.09759 (17)	0.0379 (10)	
C14	0.1044 (5)	0.6900 (3)	0.08435 (17)	0.0432 (10)	
H14	0.1310	0.7181	0.0515	0.052*	
C15	−0.2488 (4)	0.6110 (3)	0.15519 (18)	0.0492 (11)	
H15A	−0.2963	0.6022	0.1203	0.059*	
H15B	−0.2955	0.6659	0.1725	0.059*	
C16	−0.2467 (4)	0.4062 (3)	0.15006 (17)	0.0435 (10)	
H16A	−0.2715	0.3461	0.1679	0.052*	
H16B	−0.3084	0.4120	0.1181	0.052*	
S1	0.41002 (13)	0.56115 (8)	0.05360 (5)	0.0539 (3)	
S2	−0.28742 (12)	0.50583 (8)	0.19606 (5)	0.0497 (3)	

### Atomic displacement parameters (Å^2^)

**Table:**

	*U*^11^	*U*^22^	*U*^33^	*U*^12^	*U*^13^	*U*^23^
Br1	0.0655 (3)	0.0599 (3)	0.0360 (3)	−0.0119 (2)	−0.0004 (2)	0.0092 (2)
Br2	0.0722 (4)	0.0703 (4)	0.0490 (3)	0.0220 (3)	−0.0132 (2)	0.0041 (3)
C1	0.034 (2)	0.030 (2)	0.033 (2)	−0.0036 (17)	0.0022 (16)	−0.0072 (17)
C2	0.037 (2)	0.033 (2)	0.0276 (19)	−0.0034 (17)	0.0013 (16)	0.0008 (16)
C3	0.039 (2)	0.032 (2)	0.038 (2)	0.0051 (18)	−0.0051 (18)	−0.0020 (18)
C4	0.035 (2)	0.028 (2)	0.046 (2)	0.0032 (18)	0.0047 (17)	−0.0039 (18)
C5	0.050 (3)	0.039 (2)	0.030 (2)	0.006 (2)	0.0070 (18)	−0.0010 (18)
C6	0.032 (2)	0.043 (2)	0.035 (2)	0.0051 (19)	−0.0032 (17)	−0.0034 (19)
C7	0.034 (2)	0.046 (3)	0.072 (3)	0.004 (2)	0.014 (2)	0.002 (2)
C8	0.040 (3)	0.051 (3)	0.067 (3)	−0.006 (2)	0.016 (2)	−0.010 (2)
C9	0.039 (2)	0.027 (2)	0.047 (2)	−0.0050 (18)	0.0051 (19)	−0.0080 (18)
C10	0.042 (3)	0.041 (2)	0.042 (2)	0.003 (2)	−0.0049 (19)	−0.009 (2)
C11	0.039 (2)	0.036 (2)	0.031 (2)	−0.0020 (18)	0.0074 (17)	−0.0042 (17)
C12	0.036 (2)	0.033 (2)	0.034 (2)	0.0043 (18)	0.0072 (17)	−0.0033 (17)
C13	0.045 (2)	0.031 (2)	0.038 (2)	0.0117 (19)	−0.0023 (18)	−0.0021 (18)
C14	0.055 (3)	0.034 (2)	0.040 (2)	0.002 (2)	0.011 (2)	−0.0012 (19)
C15	0.038 (2)	0.054 (3)	0.056 (3)	0.010 (2)	0.007 (2)	−0.004 (2)
C16	0.033 (2)	0.048 (3)	0.049 (2)	−0.005 (2)	0.002 (2)	−0.006 (2)
S1	0.0477 (7)	0.0482 (7)	0.0658 (8)	−0.0043 (6)	0.0279 (6)	−0.0069 (6)
S2	0.0453 (7)	0.0557 (7)	0.0481 (6)	−0.0039 (6)	0.0205 (5)	−0.0097 (6)

### Geometric parameters (Å, °)

**Table:**

Br1—C2	1.905 (4)	C8—H8A	0.9700
Br2—C13	1.914 (4)	C8—H8B	0.9700
C1—C6	1.379 (5)	C9—C10	1.369 (6)
C1—C2	1.381 (5)	C9—C14	1.387 (6)
C1—C16	1.519 (5)	C10—C11	1.384 (5)
C2—C3	1.403 (5)	C10—H10	0.9300
C3—C4	1.370 (5)	C11—C12	1.377 (5)
C3—H3	0.9300	C11—H11	0.9300
C4—C5	1.398 (5)	C12—C13	1.377 (5)
C4—C7	1.508 (5)	C12—C15	1.514 (6)
C5—C6	1.364 (5)	C13—C14	1.370 (6)
C5—H5	0.9300	C14—H14	0.9300
C6—H6	0.9300	C15—S2	1.809 (5)
C7—S1	1.800 (4)	C15—H15A	0.9700
C7—H7A	0.9700	C15—H15B	0.9700
C7—H7B	0.9700	C16—S2	1.827 (4)
C8—C9	1.498 (6)	C16—H16A	0.9700
C8—S1	1.811 (4)	C16—H16B	0.9700
			
C6—C1—C2	116.1 (3)	C10—C9—C8	121.3 (4)
C6—C1—C16	119.9 (4)	C14—C9—C8	120.5 (4)
C2—C1—C16	123.8 (3)	C9—C10—C11	121.1 (4)
C1—C2—C3	121.7 (3)	C9—C10—H10	119.5
C1—C2—Br1	120.9 (3)	C11—C10—H10	119.5
C3—C2—Br1	117.4 (3)	C12—C11—C10	121.4 (4)
C4—C3—C2	120.4 (4)	C12—C11—H11	119.3
C4—C3—H3	119.8	C10—C11—H11	119.3
C2—C3—H3	119.8	C11—C12—C13	116.2 (3)
C3—C4—C5	117.9 (4)	C11—C12—C15	121.2 (4)
C3—C4—C7	120.1 (4)	C13—C12—C15	122.5 (4)
C5—C4—C7	122.0 (4)	C14—C13—C12	123.2 (4)
C6—C5—C4	120.3 (4)	C14—C13—Br2	116.9 (3)
C6—C5—H5	119.9	C12—C13—Br2	119.8 (3)
C4—C5—H5	119.9	C13—C14—C9	119.5 (4)
C5—C6—C1	123.2 (4)	C13—C14—H14	120.2
C5—C6—H6	118.4	C9—C14—H14	120.2
C1—C6—H6	118.4	C12—C15—S2	117.8 (3)
C4—C7—S1	117.8 (3)	C12—C15—H15A	107.9
C4—C7—H7A	107.9	S2—C15—H15A	107.9
S1—C7—H7A	107.9	C12—C15—H15B	107.9
C4—C7—H7B	107.9	S2—C15—H15B	107.9
S1—C7—H7B	107.9	H15A—C15—H15B	107.2
H7A—C7—H7B	107.2	C1—C16—S2	113.0 (3)
C9—C8—S1	114.2 (3)	C1—C16—H16A	109.0
C9—C8—H8A	108.7	S2—C16—H16A	109.0
S1—C8—H8A	108.7	C1—C16—H16B	109.0
C9—C8—H8B	108.7	S2—C16—H16B	109.0
S1—C8—H8B	108.7	H16A—C16—H16B	107.8
H8A—C8—H8B	107.6	C7—S1—C8	103.3 (2)
C10—C9—C14	118.0 (4)	C15—S2—C16	103.2 (2)
			
C6—C1—C2—C3	6.1 (5)	C9—C10—C11—C12	−2.1 (6)
C16—C1—C2—C3	−168.6 (4)	C10—C11—C12—C13	−4.2 (6)
C6—C1—C2—Br1	−174.1 (3)	C10—C11—C12—C15	172.1 (4)
C16—C1—C2—Br1	11.2 (5)	C11—C12—C13—C14	6.0 (6)
C1—C2—C3—C4	−1.9 (6)	C15—C12—C13—C14	−170.3 (4)
Br1—C2—C3—C4	178.3 (3)	C11—C12—C13—Br2	−176.7 (3)
C2—C3—C4—C5	−4.0 (6)	C15—C12—C13—Br2	7.0 (5)
C2—C3—C4—C7	174.8 (4)	C12—C13—C14—C9	−1.4 (6)
C3—C4—C5—C6	5.7 (6)	Br2—C13—C14—C9	−178.8 (3)
C7—C4—C5—C6	−173.1 (4)	C10—C9—C14—C13	−5.1 (6)
C4—C5—C6—C1	−1.4 (6)	C8—C9—C14—C13	169.4 (4)
C2—C1—C6—C5	−4.4 (5)	C11—C12—C15—S2	−28.6 (5)
C16—C1—C6—C5	170.4 (4)	C13—C12—C15—S2	147.5 (3)
C3—C4—C7—S1	−141.7 (3)	C6—C1—C16—S2	−100.7 (4)
C5—C4—C7—S1	37.1 (5)	C2—C1—C16—S2	73.7 (5)
S1—C8—C9—C10	104.9 (4)	C4—C7—S1—C8	70.3 (4)
S1—C8—C9—C14	−69.4 (5)	C9—C8—S1—C7	−64.4 (4)
C14—C9—C10—C11	6.8 (6)	C12—C15—S2—C16	−74.8 (3)
C8—C9—C10—C11	−167.6 (4)	C1—C16—S2—C15	62.9 (3)
